# Debridement of Diabetic Foot Ulcers

**DOI:** 10.1089/wound.2021.0016

**Published:** 2022-09-15

**Authors:** David Dayya, Owen J. O'Neill, Tania B. Huedo-Medina, Nusrat Habib, Joanna Moore, Kartik Iyer

**Affiliations:** ^1^ Division of Undersea and Hyperbaric Medicine, Department of Surgery, Phelps Hospital Northwell Health, Sleepy Hollow, New York, USA.; ^2^ Department of Allied Health Sciences, University of Connecticut, Storrs, Connecticut, USA.; ^3^ Department of Community Medicine, University of Connecticut, Storrs, Connecticut, USA.; ^4^ Department of Emergency Medicine, SUNY – Upstate Medical University, Syracuse, New York, USA.; ^5^ Department of Family Medicine, University of Vermont College of Medicine, Burlington, Vermont, USA.; ^6^ Department of Medicine, Greenwich Hospital, Greenwich, Connecticut, USA.; ^7^ Department of Medicine, Norwalk Hospital, Norwalk, Connecticut, USA.; ^8^ Department of Medicine, New York Medical College, Valhalla, New York, USA.

**Keywords:** debridement, diabetes, dressings, foot ulcers, public health

## Abstract

Diabetic foot ulcerations have devastating complications, including amputations, poor quality of life, and life-threatening infections. Diabetic wounds can be protracted, take significant time to heal, and can recur after healing. They are costly consuming health care resources. These consequences have serious public health and clinical implications. Debridement is often used as a standard of care. Debridement consists of both nonmechanical (autolytic, enzymatic) and mechanical methods (sharp/surgical, wet to dry debridement, aqueous high-pressure lavage, ultrasound, and biosurgery/maggot debridement therapy). It is used to remove nonviable tissue, to facilitate wound healing, and help prevent these serious outcomes. What are the various forms and rationale behind debridement? This article comprehensively reviews cutting-edge methods and the science behind debridement and diabetic foot ulcers.

**Figure:**
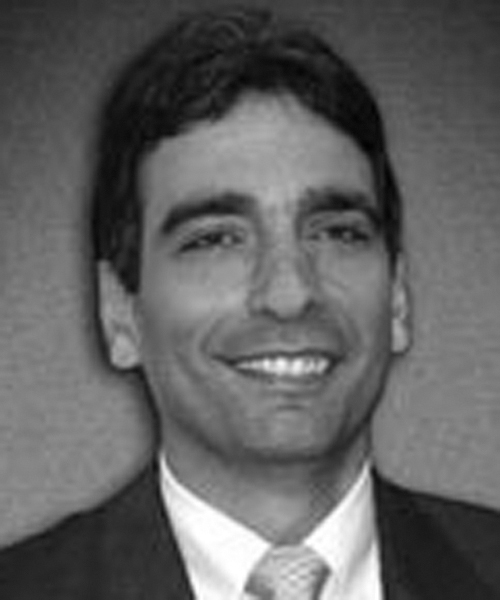
David Dayya, DO, PhD, MPH

## Scope and Significance

There are a range of therapeutic options available to health care providers for the prevention and treatment of diabetic foot ulcer(s) (DFU). This comprehensive review focuses on the subject of debridement of DFU, a widely used method to remove devitalized tissue usually occurring in the feet of diabetics. These ulcers place diabetics at higher risk for infections, amputations, and disability resulting in poor quality of life and premature mortality. It is estimated that 15–34% of diabetics will develop foot ulcers in their lifetime. Since 9.4% of the population is afflicted with diabetes mellitus (DM), the number of DFU treated is staggering.

## Translational Relevance

Devitalized tissue in a DFU acts as a barrier to healing by serving as a nidus for infection and impeding the migration of cells required in the cellular regeneration of the DFU. The chronic nonhealing DFU becomes stagnant in the inflammatory phase of healing. DFUs critically colonized with organisms and devoid of normal blood supply promote an inflammatory phase environment. This inflammatory phase of healing is evidenced by an abundance of inflammatory cells and inflammatory mediators. This inflammatory phase includes an increase in the enzyme matrix metalloprotease and inflammatory cytokines that facilitate inflammatory cellular opsonization and chemotaxis. This phase of healing attracts additional inflammatory cells and continues this repetitive detrimental cycle.

## Clinical Relevance

Understanding the complicating factors that delay wound healing at the mechanistic level of healing, including at the biochemical, cellular, and tissue level warrants the need to remove devitalized tissue from a DFU at the clinical level. Debridement helps limit the growth of pathologic organisms and tempers the inflammatory response that stagnates the DFU in the inflammatory phase of healing. Debridement effectively returns the wound to the initial acute wound phase of healing, or the hemostasis/coagulation phase of healing. Debridement promotes the progression of the DFU to advance through the stages of wound healing from the hemostasis/coagulation phase through the maturation phase of healing. The removal of devitalized tissues is critical in promoting angiogenesis, vasculogenesis, and the development of granulation tissue, which facilitates healing in an accelerated time frame and prepares the wound bed for additional intervention measures. There are a variety of debridement methods that are broadly grouped into two primary categories, including nonmechanical and mechanical debridement. Our review focuses on the science surrounding debridement of DFUs and both categories of debridement modalities.

## Introduction

The DFU is defined in accordance with the International Working Group on the Diabetic Foot (IWGDF) as a break of the skin of the foot that includes minimally the epidermis and part of the dermis, in a person with DM, this may be associated with infection, ulceration, or destruction of tissues of the foot associated with neuropathy and/or peripheral artery disease in the lower extremity.^1^ The ulcer is the result of a break in the dermal barrier, with subsequent erosion of underlying subcutaneous tissue. In severe cases, the breach may be extended to muscle and bone. The progression to ulceration may be attributed to an impaired arterial blood supply, neuropathy, musculoskeletal deformities, or a combination of these factors.^2^ If the process of wound healing is impaired and the wound progresses, then the risks of infection, amputation, morbidity, and mortality increase.^3^

Foot ulceration affects 15–34% of diabetics at some point in their lives.^4–6^ The prevalence of diabetes is estimated to include 7% (4.8 million) in the United Kingdom, 9.4% (30.3 million) in the United States, and 7% (366 million) of the world's population.^5,7^ These figures suggest the prevalence of DFUs afflict up to 1.6 million in the United Kingdom, 10.3 million in the United States, 124 million of the world's population. Debridement is regarded as an effective intervention to accelerate ulcer healing and to decrease the risk of serious complications.^8^

### Global data reports

In 2009 the International Working Group on the Diabetic Foot (IWGDF) began its efforts to produce consensus guidelines on the diabetic foot.^9^ In 2011 the IWGDF estimated that of the worldwide 7.0% (366 million) of individuals with diabetes, 80% (292 million) reside in developing countries.^9^

The IWGDF 2012 estimated that by the year 2030 there will be 8% (552 million) individuals globally who are afflicted with diabetes (Type 1/Type 2) in the adult population.^10^ Annually greater than 1 million people undergo a limb amputation, or one amputation occurs every 30 s.^9^ The prevalence of DFUs is estimated to be 19% to 34%, whereas the recurrence rate of DFUs is estimated to be 40% within a year and 65% within 3 years.^1^ The majority of amputations are preceded by a foot ulcer and the IWGDF 2019 estimates that after a major amputation up to one half of this group will die within 5 years.^1^

**Figure 1.**
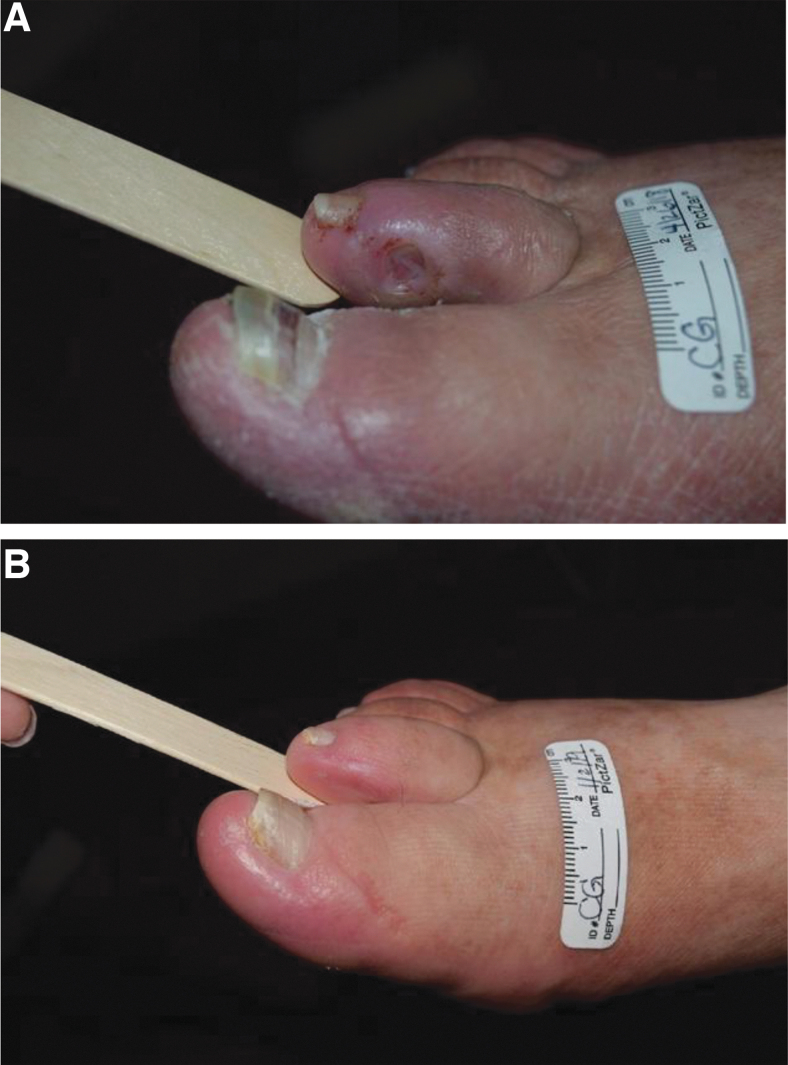
Diabetic patient before **(A)** and after **(B)** with a Wagner Grade 1 ulcer due to friction with poorly fitting shoes treated with offloading, and using a combination of sharp debridement, enzymatic debridement, and antifungal treatment to treat the onychomycosis/Tinea pedis.

**Figure 2.**
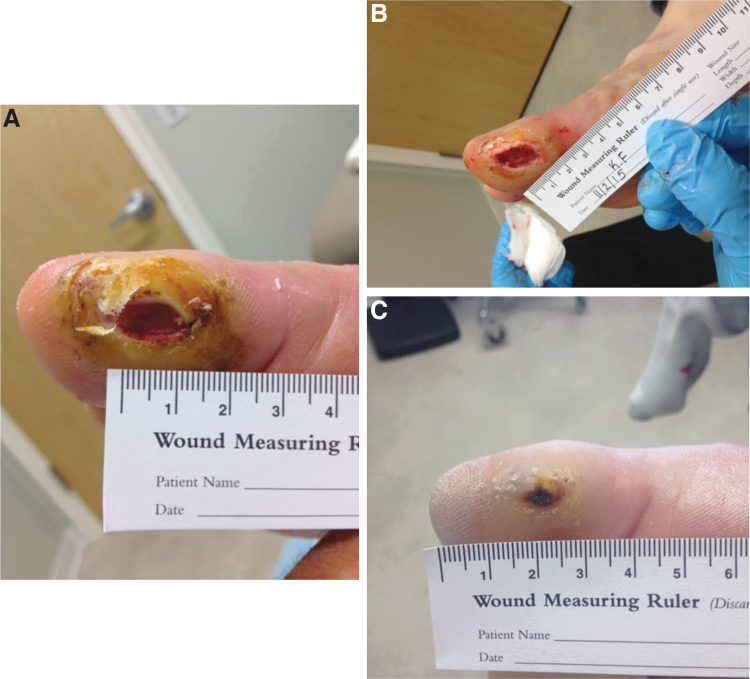
Serial images depicting measurements of Wagner grade 2 wound with progressive healing clockwise in this diabetic **(A–C)** patient using a combination of offloading and sequential debridement's lasting 12 weeks, including a combination of sharp, enzymatic, and autolytic.

**Figure 3.**
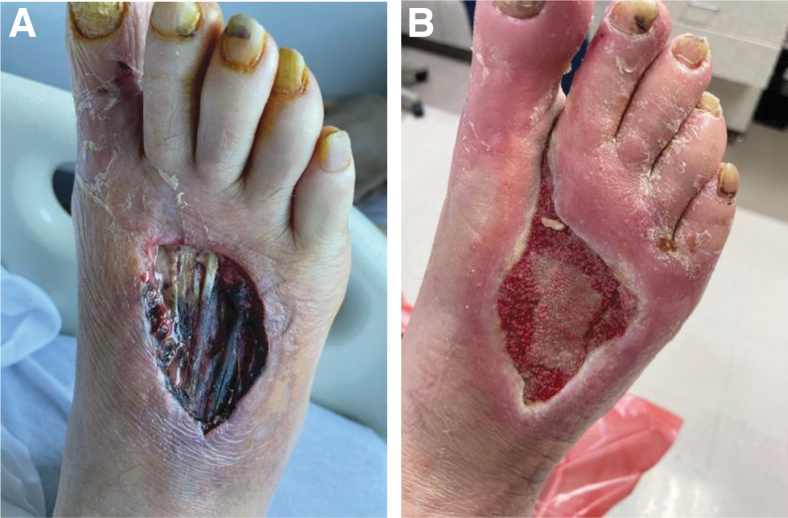
Diabetic patient with sensory impairment who stepped on a nail that penetrated his shoe and foot and did not perceive an injury until later. He developed a Wagner grade 4 wound with gangrenous involvement of the forefoot and osteomyelitis that could have resulted in amputation **(A)**. He was successfully treated with a combination of intraoperative surgical debridement, and autolytic debridement. The healthy granulation tissue appears **(B)** and the wound was amenable to receiving a graft.

The most important risk factors involved in the development of these ulcers include peripheral neuropathy, foot deformities, minor foot trauma, and peripheral arterial disease (PAD) (Figs. 1–3). Once the ulcer appears, infection and PAD are considered major causes leading to amputation. The burden of amputations in developing countries is greater than it is in developed countries. The working group estimated that ∼28–50% may progress to the point where they require amputation (major or minor).^1,9–11^

There is significant psychosocial impact in people with DFUs, including those who have required amputations. Frequently comorbid depression and a reduced quality of life result in an increase in “social isolation.”^12,13^ Chronic psychosocial stress can have immunocompromising effects.^14^ The risk of amputation is increased in these individuals living alone, and lacking in social support. Timely healing of DFU was found to be important in improving the quality of life. The working group stated that investing in diabetic foot care guidelines is one of the most cost-effective forms of health-care expenditure in addressing these psychosocial concerns among other risks.^10^

The global data by the IWGDF is contrasted below with country-specific data using United States and United Kingdom data to better compare global health data to that of industrialized countries in Europe and North America. This contrast is to help the reader better appreciate the context of global heath data from the developing world against country-specific health data from selected representative countries in the industrialized world (Table 1).

**Table 1.** Costs of treating foot ulcers and amputations

Reference	Country	Number of Patients	Costs (Year of Costing)	USD 2005 equivalent (USD 2/2021 Equivalent)^99^	Comments
Ulcers not requiring amputation					
Apelqvist *et al.* (1994)^100^	Sweden	197	SEK 51,000 (1990)	8,654 (11,935)	All ulcer types; total
Harrington *et al.* (2000)^40^	United States	400,000	USD 3,999–6 (1996)	4,982–7,821 (6,871–10,787)	Inpatient and outpatient costs
Holzer *et al.* (1998)^37^	United States	1,846^c^	USD 1,929 (1992)	2,695 (3,717)	Inpatient and outpatient costs, those >64 years. excluded
Mehta *et al.* (1999)^101^	United States	5,149	USD 900–2,600 (1995)	1,150–3,322 (1,586)	Private insurance charges; mean age 51 years.
Tennvall *et al.* (2000)^9^	Sweden	88	SEK 136,600 (1997)	18,719 (25,817)	Deep foot infection; total direct costs
Ramsey *et al.* (1999)^14^	United States	514^d^	USD 27,987 (1995)	35,758 (49,317)	Including 2 years. after diagnosis
Van Acker *et al.* (2000)^102^	Belgium	120	USD 5,227 (1993)	7,039 (9,708)	Inpatient and outpatient costs^g^
Costs of lower extremity amputations					
Apelqvist *et al.* (1994)^100^	Sweden	27	SEK 258,000 (1990)	43,778 (60,379)	All ulcer types; minor LEA; total direct costs
Apelqvist *et al.* (1994)^100^	Sweden	50	SEK 390,000 (1990)	66,176 (91,270)	All ulcer types; major LEA; total direct costs
Ashry *et al.* (1998)^22^	United States	5,062	USD 27,930 (1991)	39,891 (55,018)	Hospital charges only
Holzer *et al.* (1998)^37^	United States	504^c^	USD 15,792 (1992)	22,062 (30,428)	Gangrene/amputation, those >64 years. excluded
van Houtum *et al.* (1995)^103^	Netherlands	1,575^e^	NLG 28,433 (1992)	19,052 (26,277)	Hospital costs only
Panayiotopoulos *et al.* (1997)^54^	United Kingdom	20	GBP 15,500 (1994–95)	33,587 (49,082)	Inpatient and prostheses costs (46% diabetics)
Tennvall *et al.* (2000)^9^	Sweden	77	SEK 261,000 (1997)	35,767 (49,330)	Deep infection; minor LEA; total direct costs
Tennvall *et al.* (2000)^9^	Sweden	19	SEK 234,500 (1997)	32,136 (44,322)	Deep infection; major LEA; total direct costs
Van Acker *et al.* (2000)^102^	Belgium	7	USD 18,515 (1993)	24,933 (34,388)	Inpatient and outpatient costs; minor LEA
Van Acker *et al.* (2000)^102^	Belgium	9	USD 41,984 (1993)	56,538 (77,977)	Inpatient and outpatient costs; major LEA

For comparison of the results, costs were first adjusted for inflation to 2005 prices with the consumer price index f and then converted to USD with the appropriate currency exchange rate for 2005. (Please note: U.S. Department of Labor and Statistics Inflation Calculations were used and are in brackets below the 2005 costs to make the conversion to compare to 2/2021 cost equivalency.)

Please note that the above table is a compilation of studies investigating costs associated with treating leg and foot wounds in diabetics was developed by the IWGDF; however, these costs may include costs incurred for treating wounds other than diabetic foot ulcers, but can also be associated with diabetics such as ischemic ulcers, pressure ulcers, and venous stasis ulcers.

A table displaying data from IWGDF 2012 (Reproduced here with permission from the IWGDF).

^a^ Based on data from observational studies.

^b^ Based on data from databases and other secondary sources.

^c^ Number of episodes.

^d^ Includes 80 amputations.

^e^ Number of hospitalizations.

^f^ Outpatient costs are direct medical costs incurred by patients receiving ambulatory care.

^g^ Inpatient costs are direct medical costs incurred as a result of care rendered in the course of hospitalization.

IWGDF, International Working Group on the Diabetic Foot; LEA, lower extremity amputation; Major, amputation above the ankle; Minor, amputation below the ankle; NA, not applicable.

The diabetes epidemic includes 9.4% (30.3 million) of children and adults, in the U.S. population, ∼1 in 11 individuals.^7^

A total of 24.2 million people are diagnosed, 7.1 million people are undiagnosed, and ∼84.1 million adults are living with prediabetes.^7,15^ This includes over 114.4 million individuals with some stage of diabetes, ∼37% of the U.S population, which correlates with rising rates of obesity and hypertension. There were 1.4 million incident cases of diabetes diagnosed in people 18 years of age and older in 2015.^15^ The disease burden varies among sex, race, and ethnicity, including: 13.3% of men, 10.8% women, 7.4% of non-Hispanic Caucasians, 8% of Asian Americans, 12.7% of non-Hispanic Blacks, 12.1% of Hispanics, and 15.1% of Native Americans.^7^

The annual death toll includes 270,702 deaths due to DM, exceeding HIV/AIDS and Breast Cancer combined.^7^ The diabetes epidemic is the number one cause of blindness and vision disability or 11.7%.^7^ It is the leading cause of kidney failure accounting for 37% (288,451) of new cases per year, and as in the United Kingdom is the leading cause of amputations in the United States.^7,16^

Surgical amputations in the United States have reached staggering levels among diabetics, 70% (130,000) of total amputations occur in diabetics.^7^ The prevalence of DFUs over the last decade has ranged and is estimated to be ∼5–8% of the diabetic population.^15,17^ The prevalence of DFUs in 32 developing and developed countries ranged from 1.5% in Australia to a high of 16.6% in Belgium.^18^ Approximately 15–34% of diabetics are expected to develop wounds in their lifetime.^4–6,19^ Based on pathophysiology irrespective of diabetes status, 82% of amputations are due to vascular disease (including diabetics), 22% due to trauma, 4% due to congenital causes, and 4% due to tumors.^20,21^ Approximately 1.6 million people are living with amputations in the United States and ∼185,000 lower limb amputations are performed each year from all causes in the United States.^15^

Among diabetics ∼55% of all amputations occur in people over the age of 65.^22,23^ Amputation rates are higher in males than they are in females, 12% versus 10.8%, respectively.^24^African Americans with diabetes have a 1.5 to 2.5 times greater rate of amputation than their Caucasian diabetic counterparts.^7,25^ Poor circulation combining macroarterial and/or microarterial occlusive disease are the main causes of amputation and account for over half of all amputations that occur among diabetics.^7,15,26^ Major amputations (above knee/below knee) are a marker for increased mortality with an estimated increase in 5-year mortality as high as 61–74% after a major amputation.^27,28^

Approximately 7.1% (4.8 million) of the UK population are estimated to have diabetes, which is increased from 2% of the population almost a decade earlier.^5^ Around 8% of diabetics have Type 1 diabetes and 90% have Type 2 diabetes.^29^ Foot ulceration is thought to affect 10–25% of people with diabetes at some time in their lives.^30^ In the United Kingdom, people with diabetes are 30 times more likely to undergo an amputations than people without diabetes.^29^ In the United Kingdom, there is up to a 70% increase in risk of people dying within 5 years of having an amputation due to diabetes.^31^ Diabetes accounts for approximately one-half of all limb amputations in the United Kingdom.^5,32^

The data reported in the United Kingdom on DM, amputations, and DFU remain significantly elevated as compared with those reported previously in 1997 and 2009.^33–35^ The World Health organization reported that 8% of males and 7% of females had raised blood glucose in the United Kingdom based on the 2014 data estimates.^36^ Additionally in 2016, 29% of males and 30% of females were obese, while 22% of males and 19% of females had raised blood pressure.^36^

These risk factors are collectively referred to as metabolic syndrome and are driving the rising trend in diabetes, DFUs, and the associated complications, including amputations.^37^ Metabolic syndrome is defined according to the International Diabetes Federation (IDF) as^38^:

(1) Central obesity with waist circumference with ethnicity specific values plus ANY 2 of the following four factors.
(i) Raised triglycerides ≥150 mg/dL, or specific treatment for this lipid abnormality.
(ii) Reduced HDL Cholesterol <40 mg/dL in males OR <50 mg/dL in females OR specific treatment for this lipid abnormality.
(iii) Raised blood pressure ≥130 mm Hg systolic OR ≥85 mm Hg diastolic OR treatment of previously diagnosed hypertension.
(iv) Raised fasting plasma glucose >100 mg/dL OR previously diagnosed Type 2 DM.

### Estimated costs

#### Global cost estimates

The IWGDF has issued a report on the cost of DFUs and amputations (Table 1).

Foot-related problems may use 12–15% of health care resources for diabetes in the developed world, whereas in developing countries this may be as high as 40% (see Table 1 above).^1,9,10^

Global estimates of direct and indirect costs include a global economic burden that will increase from $1.3 trillion to $2.2 trillion by 2030 in U.S currency. This increase in costs as a shared global GDP is estimated to rise from 1.8% in 2015 to a maximum of 2.2% by 2030.^39^

The estimated total cost of diabetes, including direct and indirect costs in the United States in 2017 was $327 billion.^40,41^ This included a direct cost of $237 billion and an indirect cost of $90 billion. Direct costs include medical costs, such as inpatient visits, emergency department visits, outpatient visits, prescription drugs, medical equipment, and home health services. Whereas indirect costs primarily relate to nonmedical costs, such as lost productivity, wages, work absence, and travel expenses, associated with receiving treatment.

The peak age-range for amputations is between 41 and 70 years, a time period of prime working age and productivity for adults.^24^ This poses a significant health challenge to our workforce since amputations can result in permanent impairment often qualifying an individual for disability benefits resulting in lost wages and productivity.^42^ This poses a significant stress on the family unit. It imposes an economic burden upon society at large in providing for impaired and disabled individuals. The rate of amputations is rising and these factors are directly contributing to this alarming trend.^7,15,26^

The U.S. Government estimates for the 2017 GDP portion allocated for direct health care costs was $2.2 trillion or 16% of the GDP.^43^ Chronic diseases, including heart disease, stroke, cancer, and diabetes, cause 7 out of 10 deaths and are responsible for 75% of the $2 trillion spent on health care.^44^ In comparison, the direct and indirect costs for diabetes in 2007 was ∼10% of 2.2 trillion dollars. Up to 15% of costs for DM in the developed world is estimated to be allocated for foot-related problems, ∼33 billion dollars in the United States.^10,45^

The UK National Health Service (NHS) spends an estimated £14 billion per year on diabetes or 11.7% of the NHS budget.^46^ Total direct and indirect costs for diabetes in the United Kingdom is £23.7 billion per year.^5^ A report published in 2019 estimates that the British NHS spends up to £1 billion spent on foot ulcers or amputations each year.^46,47^

### Definition and description of the condition—DFUs

#### Wound progression

The DFU is considered multifactorial in its etiology. Wound repair and closure will help re-establish hemostasis, preserving the barrier function of the skin to prevent infection, and maintaining the overall protective role of skin.

Wound healing progresses through the following phases: (1) Hemostasis/Coagulation phase, (2) Inflammatory phase, (3) Proliferative phase, (4) Maturation/Remodeling phase.^3,48,49^

Problems with wound healing are considered multifactorial in diabetics. These prognostic factors may include the following: vascular insufficiency/PAD, peripheral neuropathy (sensory/motor/autonomic), immunosuppression, and critical colonization/infection.

These problems may be more common in the presence of nonviable tissue (contributing to increased risk of infection and delayed wound healing), smoking (contributory to the risk of peripheral arterial insufficiency), and poor nutritional status (inadequate protein/nutrients required for wound healing). It is believed that these combined problems contribute to the wound stagnating within the inflammatory phase of the healing process. The development of a wound involves minor soft tissue injury or insult compounded by these factors. The trauma can be the result of friction, mechanical shearing forces, direct pressure, or penetrating tissue injury, including sharp or blunt trauma.^3,48,49^

#### Vascular insufficiency

Diseases of blood vessels, including arterial and venous, whether macrovascular or microvascular are a major cause of complications in diabetes and can complicate wound healing of DFUs.^50^ The Framingham study reported that more than 50% of men and women with diabetes had absent foot pulses.^51^ The Framingham study is in its third generation of participants and comprises a total of 4,095 people.^52^

PAD tends to occur at younger ages in people with diabetes and is believed to involve smaller blood vessels and capillaries. Reports from United States, United Kingdom, and Finland concur that PAD is a major contributory factor in the pathogenesis of foot ulceration and subsequent major amputations.^53–56^

Impaired blood flow can occur at both levels of the microarterial and macroarterial circulation in diabetics compounding the problem of delayed wound healing from inadequate tissue oxygenation and nutrients. Microcirculation involvement includes the occlusion of small blood vessels and capillaries, whereas macroarterial insufficiency is defined as the occlusion of medium- and large-sized blood vessels.

Hemodynamically significant macrovascular arterial insufficiency is considered an advanced stage of PAD, which may warrant surgical revascularization procedures.^57^ These vascular occlusions and the resulting wound hypoxia poses a major risk factor in the development of nonhealing problem wounds.^3,10,58^ A host of considerations are believed to compound vascular insufficiency, which restricts the delivery of oxygen and nutrients required for adequate wound healing, immune function, and can increase susceptibility of coinfections. These considerations may include nutritional status, cardiovascular insufficiency, hydration status, psychosocial factors, smoking and alcohol history, patient compliance, socioeconomic status, availability of ancillary treatment modalities, proficiency and expertise of the health care provider involved in the wound care, the type of wound, and the presence of wound occurrence from multifactorial wound mechanisms, the age of the patient, and possibly the type of debridement method provided to the patient for removal of nonviable tissue from the wound bed and the periwound.^3,9,10,59,60^

Venous insufficiency may delay wound healing due to edema that increases the diffusion distance for oxygen to travel from the arterial circulation across the tissue to the wound bed, and by compromising capillary diffusion through increased tissue hydrostatic impeding capillary flow.^61^

### Neuropathy (sensory, motor, autonomic)

Impairment of nerve function is an important and frequent complication of diabetes. All types of nerve fibers can be affected, including motor, sensory, and autonomic nerve fibers, and their functions. Impaired nerve function in the foot is common in people with diabetes although the person themselves may be unaware of its presence. Neuropathy remains one of the major factors leading to the development of foot ulceration in people with diabetes.^62^

Approximately 60–70% of diabetics have neurologic disease, most often a peripheral neuropathy involving the lower extremities.^7,15,26^

This microvascular disease component is believed to cause occlusion within the vasa nervorum, which provides the blood supply to the nerves possibly due to the direct cytotoxic effect of the hyperglycemia.^63^ This form of microvascular occlusive disease contributes to the development of peripheral neuropathy. Since diabetic neuropathy involves motor, sensory, and autonomic nerve fibers, the pathologic deficits may include the deformed, insensate, and dry cracking foot.

#### Sensory neuropathy

Damage to the nerves responsible for conducting afferent sensory perception from the foot renders the foot insensitive to temperature, vibration, pressure, and pain. These are referred to as sensory neuropathy. The loss of sensation means that relatively minor injuries often go undetected and reinjury or repetitive cumulative trauma can result in a wound that progressively worsens in severity. Repetitive cumulative trauma can result from ill-fitting shoes resulting in friction. The insensate foot, unlike a normal innervated foot, does not warn the individual to make the needed adjustments or changes required to arrest the insults responsible for wound progression and infection. The insensate foot does not result in the kind of vascular neuroregulatory changes required to supply the injured area sufficiently with oxygen and nutrients required for wound healing, which compounds the microvascular insufficiency. Sensory function is frequently tested using a 128 Hz tuning fork and Semmes-Weinstein Monofilament.^1^

#### Motor neuropathy

Denervation of muscles has direct effects on the function of the foot. The small muscles of the foot, the extensor digitorum brevis, lumbrical, and interosseous muscles are commonly affected.^64^ Paralysis of these small muscles results in the metatarsophalangeal joints becoming hyperextended and the interphalangeal joints becoming flexed.^62^ The joints initially remain mobile, but later degenerative changes occur and the joints become fixed.^1,62^

The consequence of such muscle wastage is a foot shape that increases foot pressures over bony prominences where wounds most commonly occur in diabetics (Fig. 1).^65^

#### Autonomic neuropathy

Autonomic neuropathy is thought to contribute to the pathogenesis of ulceration, neuropathic edema, and Charcot arthropathy.^1,62^

Impairment of sweating contributes to the formation of hyperkeratotic plaques and fissures in the skin. Callus (increased glycation of keratin) becomes thick, pressing on the soft tissues underneath contributing to ulceration.^66^ A callus defined as a buildup of keratinized skin in reaction to persistent pressure can exert pressure on the soft tissues of the foot.^67^ The dry cracking foot is a function of this neuropathy, anhidrosis, and impaired temperature regulation that contribute to these local effects.^10^

### Immunosuppression/critical colonization/infection

Diabetes is considered an immunocompromising condition. It has been observed that white blood cells may behave atypically in a hyperglycemic environment and do not marginate, migrate, or secrete the cytokines sufficiently that are required to combat infection.^68^ This can increase the risk of critical colonization and infection.

The immunosuppressive state that may occur in diabetics with an open wound can lead to critical colonization and infection increasing the risks of a nonhealing chronic wound.^3,10,68^ The chronicity of this condition increases the risk of methicillin-resistant *Staphylococcus aureus* (MRSA), which is among the cultured organisms found in chronic wounds and a major public health concern. Infections that have reached the deeper bony level of tissue involvement may become refractory to treatment. The patient can be at risk for life-threatening sepsis from a wound as the source of infection.^3,10^ This may warrant urgent amputation to remove the source of life-threatening sepsis.

### Pathway to ulceration

Despite the presence of the predisposing factors noted above, an uninjured foot may not develop serious problems. Physical trauma is an inciting event, that is, puncture, localized pressure, and recurrent mechanical trauma, including friction, heat, or chemical injury.^65,69^

When sensory impairment is present, a small lesion may progress because it may go unrecognized, and the source of injury not alleviated. Lack of sensation progresses to ulceration and impairment of the blood supply further delays healing. Complicating infections further increase the damage to tissues.^65^

Chronic wounds are generally defined as wounds present beyond 4 weeks without significant clinical improvement. These chronic wounds may continue to progress beyond full thickness (limited to epidermis and dermis). This progression can involve deeper tissue(s), including hypodermis, muscle, tendon, and bone. Progression of vascular compromise and infection may lead to tissue ischemia, nonviable tissue, and gangrene, ultimately leading to limb amputation.^69^

Deep-seated wound infections such as chronic osteomyelitis and significant bone destruction can become considerations in the decision to amputate limbs.^7,9,15,26,59,60^

### Common grading systems used to classify the severity of diabetic wounds and risk of amputation.

The Wagner grading system and the Texas classifications are internationally utilized grading systems.^70–72^ These grading systems were compared, and the results concluded that increasing stage, regardless of the grade, is associated with increased risk of amputation and a delay in ulcer healing time. The University of Texas system's inclusion of stage suggested it was a superior predictor of outcome.^73^ The University of Texas System grading system provides subclassifications regarding presence of infection and ischemia along with the depth of tissue involvement (Tables 2 and 3).

**Table 2.** Wagner wound grade classification system

0	1	2	3	4	5
No ulcer in a high-risk foot	Wound involving full skin thickness	Wound extending to ligament and muscle	Wound with cellulitis or abscess	Localized gangrene	Extensive gangrene involving the whole foot

**Table 3.** University of Texas wound classification system

	Grade			
Stage	0 Pre- or Postulcerative lesion completely epithelialized	1 Superficial wound not involving tendon, muscle, or bone	2 Wound penetrating to tendon or capsule	3 Wound penetrating to bone or joint
A No Infection, or ischemia	0A	1A	2A	3A
B Infection but no ischemia	0B	1B	2B	3B
C Ischemia but no infection	0C	1C	2C	3C
D Infection and ischemia are present	0D	1D	2D	3D

Critical limb ischemia refers to a threatened lower extremity mainly due to chronic ischemia. The Society for Vascular Surgery developed a lower extremity threatened limb classification system. This system is based on three major factors that include the severity of the wound, and the presence and severity of both ischemia and foot infection (Wound, Ischemia, foot Infection [WIfI]) (Tables 4 and 5). The three risk factors are graded or staged individually and are used in combination on a scale to predict the risk of amputation at 1 year and the potential benefit of revascularization.^74^ The wound grading system(s) and the WIfI classification system should be used in determining the appropriateness and indication for debridement. This influences the type of debridement method used, for example sharp debridement may not be appropriate in critical limb ischemia. Hemodynamically significant ischemia that complicates DFU may require revascularization as a prerequisite to debridement.

**Table 4.** Summary and comparison of existing diabetic foot ulcer, wound, and lower extremity ischemia classification systems

			
I. Wound II. Ischemia III. foot Infection W I fI score			
W: Wound/clinical category SVS grades for rest pain and wounds/tissue loss (ulcers and gangrene):			

Grade	Ulcer	Gangrene	
0	No ulcer	No gangrene	
Clinical description: ischemic rest pain (requires typical symptoms + ischemia grade 3); no wound.			
1	Small, shallow ulcer(s) on distal leg or foot; no exposed bone, unless limited to distal phalanx	No Gangrene	
Clinical description: minor tissue loss. Salvageable with simple digital amputation (1 or 2 digits) or skin coverage.			
2	Deeper ulcer with exposed bone, joint or tendon; generally not involving the heel; shallow heel ulcer, without calcaneal involvement	Gangrenous changes limited to digits	
Clinical description: major tissue loss salvageable with multiple (≥3) digital amputations or standard TMA ± skin coverage.			
3	Extensive, deep ulcer involving forefoot and/or midfoot; deep, full-thickness heel ulcer ± calcaneal involvement	Extensive gangrene involving forefoot and/or midfoot; full thickness heel necrosis ± calcaneal involvement	
Clinical description: extensive tissue loss salvageable only with a complex foot reconstruction or nontraditional TMA (Chopart or Lisfranc); flap coverage or complex wound management needed for large soft tissue defect			
TMA, transmetatarsal amputation			
I: Ischemia Hemodynamics/perfusion: measure TP or TcPO_2_ if ABI incompressible (>1.3)			

Grade	ABI	Ankle systolic pressure	TP, TcPO_2_
0	≥0.80	>100 mm Hg	≥60 mm Hg
1	0.6–0.79	70–100 mm Hg	40–59 mm Hg
2	0.4–0.59	50–70 mm Hg	30–39 mm Hg
3	≤0.39	<50 mm Hg	<30 mm Hg
Patients with diabetes should have TP measurements. If arterial calcification precludes reliable ABI or TP measurements, ischemia should be documented by TcPO_2_, SPP, or PVR. If TP and ABI measurements result in different grades, TP will be the primary determinant of ischemia grade. Flat or minimally pulsatile forefoot PVR = grade 3. ABI, Ankle–Brachial Index; PVR, pulse volume recording; SPP, skin perfusion pressure; TP, toe pressure; TcPO_2_, transcutaneous oximetry.			
fI:			
SVS grades 0 (none), 1 (mild), 2 (moderate), and 3 (severe: limb and/or life threatening) SVS adaptation of Infectious Diseases Society of America (IDSA) and IWGDF perfusion, extent/size, depth/tissue loss, infection, sensation (PEDIS) classifications of diabetic foot infection			

Clinical manifestation of infection	SVS	IDSA/PEDIS infection severity	
No symptoms or signs of infection	0	Uninfected	
Infection present, as defined by the presence of at least two of the following items: • Local swelling or induration • Erythema >0.5 to ≤2 cm around the ulcer • Local tenderness or pain • Local warmth • Purulent discharge (thick, opaque to white, or sanguineous secretion)	1	Mild	
Local infection involving only the skin and the subcutaneous tissue (without involvement of deeper tissues and without systemic signs as described below). Exclude other causes of an inflammatory response of the skin (*e.g.*, trauma, gout, acute Charcot neuro-osteoarthropathy, fracture, thrombosis, venous stasis) 1 Mild Local infection (as described above) with erythema >2 cm, or involving structures deeper than skin and subcutaneous tissues (*e.g.*, abscess, osteomyelitis, septic arthritis, fasciitis), and No systemic inflammatory response signs (as described below)	2	Moderate	
Local infection (as described above) with the signs of SIRS, as manifested by two or more of the following: • Temperature >38°C or <36°C • Heart rate >90 beats/min • Respiratory rate >20 breaths/min or PaCO2 < 32 mm Hg • White blood cell count >12,000 or <4,000 cu/mm or 10% immature (band) forms 3	3	Severe^a^	

Reprinted with permission from Elsevier.^74^

fI, foot Infection; TMA, transmetatarsal amputation.

**Table 5.** Risk/benefit: clinical stages by expert consensus

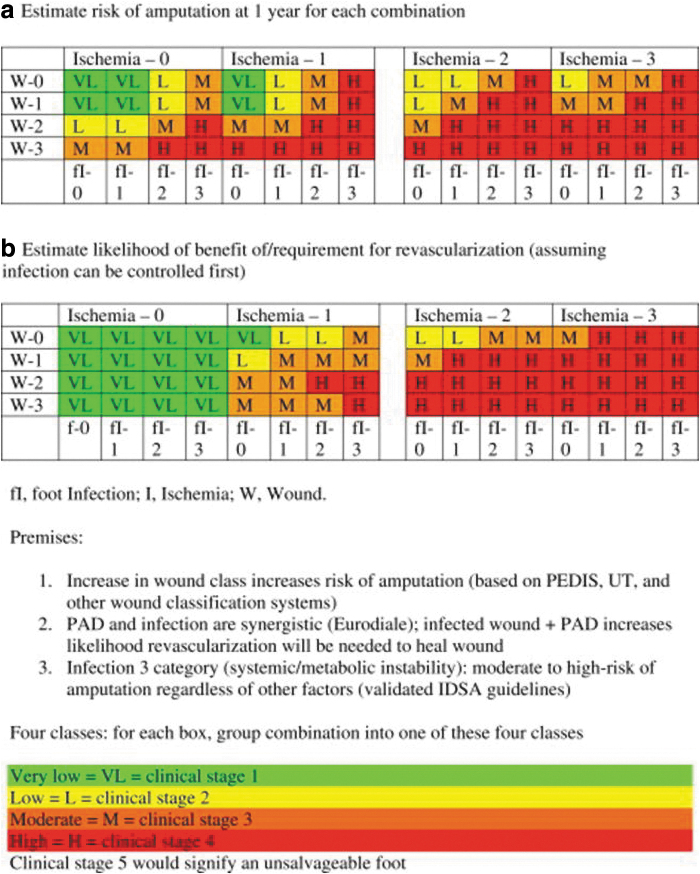

## Description of the Intervention

### Debridement as the wound care intervention of interest

Currently debridement is considered a central component of conventional wound care. This is used to remove nonviable tissue, which may pose a risk of colonization and infection. Nonviable (necrotic) tissue may impede wound healing by obstructing cellular migration across the wound inhibiting the normal development of the wound bed and prevent granulation tissue formation.^3,48,75^

Debridement enables the clinician to better gauge the size of the wound and may facilitate drainage from the wound.^3,75^ If wound culture is clinically indicated it should be obtained post-debridement. If cleansing of the wound is required prior to obtaining cultures, then saline and not anti-septic solution should be utilized to reduce false negative results for cultures taken from the wound.^3^ The editorial board of the journal Wound Source reported on 12/31/2018 that critical colonization is defined as proliferating organisms with a host response but without invasion and ≤ 105 organisms/gram of tissue. Critical colonization may present with subtle findings.

Treatment focuses on closing the wounds within the first 4–6 weeks of their development.^3^ Wounds that decrease their surface area by 20–40% within the first 4 weeks are considered to have a higher likelihood of closing.^3,48^ Desirable goals include reducing the time to complete healing, accelerating healing rates, and reducing the rates of wound recurrence.

If the wound is closed in a timely manner, the risks of complicating infections, and amputation may be prevented thus improving the patient's overall quality of life. Although applicable to DFU, this article will not address other interventions, such as negative pressure wound therapy that have multiple functions beyond debridement. See Table 6 that describes, compares, and contrasts the advantages and disadvantages, and lists the indications and contraindications for both mechanical and nonmechanical debridement methods.

**Table 6.** A Comparison of contrasting debridement methods

Debridement Methods	Description	Advantages	Disadvantages	Indications	Contraindications
Nonmechanical		Selective and Specific for nonviable tissue Convenient simple application None or minimal discomfort Less costly	Tend to be slower (days to weeks)	1. Removal of potential source of infection and sepsis, primarily nonviable tissue. 2. Removal of critically colonized tissue to decrease bacterial burden, reduce the probability of resistance from antibiotic treatment, and obtain accurate cultures. 3. Facilitate the collection of deep cultures taken postdebridement to evaluate requirements for antibiotic treatment 4. Stimulation of the wound bed to support healing and prepare for procedures, including but not limited to grafts, flaps, and to support skin substitutes.	1. Contraindications will pertain to the specific method of debridement (see below). 2. Refrain from debridement of dry and intact eschars that have no clinical evidence of underlying infection and could potentially serve as a biological dressing.
Autolytic	Relies on a dressing type that permits the wound to remain moist and facilitate autolysis of the devitalized tissue.	Selective for nonviable tissue. None or minimal discomfort. Convenient simple application Less costly	Slow process (days to weeks) May be associated with maceration of surrounding tissue. Can be colonized and complicated by infection. May be associated with maceration in surrounding tissue with highly exudative wounds. Not ideal in heavily infected wounds. Less costly	Same	If there is active infection with large amounts of devitalized tissue needing removal (*i.e.*) gangrenous tissue, a different method of debridement should be considered.
Enzymatic	Uses the application of an enzyme, such as collagenase, to help lyse nonviable tissue.	Quicker than Autolytic debridement Selective and specific for nonviable tissue. None or minimal discomfort. Convenient simple application	Slow process (days to weeks) May be associated with maceration in surrounding tissue with highly exudative wounds. Not ideal in heavily infected wounds. May be deactivated by other treatments used in wound care. Expensive	Same	1. A relative contraindication is its use in heavily infected wounds. 2. Collagenase should not be used with silver-based products or with Dakin solution.
Mechanical		Relatively quicker than nonmechanical debridement	May be selective or nonselective depending on specific method used. May be less convenient. May be associated with more pain. Expensive	1. Removal of potential source of infection and sepsis, primarily necrotic tissue. 2. Removal of critically colonized tissue to decrease bacterial burden, reduce the probability of resistance from antibiotic treatment, and obtain accurate cultures. 3. Facilitate the collection of deep cultures taken postdebridement, to evaluate requirements for antibiotic treatment 4. Stimulation of the wound bed to support healing and prepare for future procedures, including but not limited to grafts, flaps, and to support skin substitutes. 5. May require local or general anesthesia. which is associated with inherent risk.	1. May vary depending on modality of mechanical debridement used (see below). 2. The presence of granulation tissue covering the wound bed and the absence of devitalized tissue. 3. Inadequate pain control. 4. Poor tissue perfusion and hypoxia surrounding the anatomical region affected. 5. Intact eschar with no gross clinical evidence of an underlying infection that could potentially serve as a biological dressing.
Sharp/surgical	Uses a form of sharp instrument, such as a scalpel or scissor, to mechanically remove devitalized tissue in an ambulatory or operative setting.	Quick Specific Painful Expensive	More postprocedure pain. Expensive especially if requiring operative room debridement.	Same	1. Operative debridement requires appropriate surgical risk stratification of the individual patient. 2. Patients with intact eschar and no clinical evidence of an underlying infection should not be debrided when the intact eschar functions as a biological covering for the underlying skin defect.
Wet to dry	Utilizes saline-moistened gauze that is allowed to dry and is then removed with the nonselective mechanical removal of devitalized tissue.	Quick Nonspecific Painful May be associated with more cost as compared with some nonmechanical debridement methods.	Nonspecific nonselective removal of granulation tissue. Postprocedure pain or discomfort.	Same	Same as discussed in mechanical debridement.
Aqueous high-pressure lavage irrigation, or whirlpool	Utilizes high-pressure irrigation, which can be done manually using a 20-mL syringe and an 18-gauge angiocatheter delivering 12 psi or high-pressure jet stream of fluid either from a whirlpool or other mechanical irrigation device.	Quick Suited for larger wounds.	Nonspecific Nonselective Less specific possibility of cross-contamination of other wounds and infection. May be associated with postprocedure pain or discomfort. May require immersion. Expensive	Same	Same as discussed in mechanical debridement. Risk of cross-contamination in the presence of multiple wounds
Ultrasound debridement	Utilizes a method of cavitation to generate sound energy from a handheld instrument that through mechanical means dislodges and removes devitalized tissue.	Quick Specific	May be associated with postprocedure pain of discomfort. Risk of exposure to aerosolized organisms and debris to the health care provider from the wound. Expensive	Same	Contraindications: Same as discussed in mechanical debridement.
Biosurgery Maggot debridement therapy	This method utilizes maggots that are applied in the larva stage and consume devitalized tissue selectively and are removed usually within 3 days.	Relatively quick Ultraspecific	May be associated with minor pain or discomfort. Patient reluctance, psychological factors.	Same	1. Abdominal wound contiguous with the intraperitoneal cavity. 2. Pyoderma gangrenosum with immunosuppression therapy. 3. Wounds in close proximity to areas afflicted by septic arthritis.

Nonmechanical debridement (Table 6)

#### Enzymatic debridement

This involves the use of exogenous enzyme products that digest the nonviable tissue, as opposed to exclusively relying on endogenously produced wound enzymes such as matrix metalloproteinases that provide autolytic debridement (Table 6).^75^

#### Autolytic debridement

This approach involves keeping the wound moist, which may facilitate the endogenous enzymes produced by the wound itself to auto-digest or “self-digest” nonviable tissue.^75^ The use of agents such as hydrogel facilitates moist wound healing and allows endogenous locally produced enzymes to digest the nonviable tissues. Many topical agents that are applied directly to skin facilitate autolytic debridement such as topical antimicrobials even though they are also used to treat critical colonization and localized wound infections.

The ability of a variety of topical agents to maintain a moist wound environment permits concurrent autolytic debridement irrespective of the other functions of the topical agent used. Other dressings that facilitate autolytic debridement include Alginates, Hydrocolloids, Foam, Film, and Honey. Moist saline gauze is commonly used and has served as a control condition or standard form of debridement in studies.^3,75^

Mechanical debridement

This method uses mechanical energy such as surgical debridement, high-pressure saline irrigation, whirlpool, wet to dry saline dressings, ultrasound, or jet lavage.^75^ The nonselective nature of these forms of debridement can remove granulation tissue that is produced during the proliferative phase of wound healing.^48^ See Table 6 that describes, compares, and contrasts advantages and disadvantages, indications, and contraindications of mechanical debridement methods.

(1) *Sharp surgical debridement*—This may be performed either in the inpatient or outpatient settings. It may be done in the operating room if an extensive debridement is required or as an outpatient “office” surgical procedure when the debridement is less extensive and superficial. Ultimately the decision on what setting in which to perform the debridement is based both upon the patient's comfort level, the degree of anesthesia required, and how extensive a debridement procedure is required.^75^
Expert opinion in sharp surgical debridement has generally dictated that the nonviable and necrotic tissue should be removed and debrided down to bleeding tissue, in effect creating a “new acute wound.”^59^ This restarts the phases of wound healing from the beginning and is not possible without injuring healthy tissue in the process of attempting to remove nonviable tissue. This dissection process can be time-intensive and is considered semiselective or nonselective.^75^ The injury of healthy tissue results from the delicate task of separating viable from nonviable tissue using standard sharp/blunt dissection instruments, that is, scalpels and curettes.
Gross dissection using instruments classified as blunt are not capable of ultraselective microdissection even in the hands of the most skilled health professionals. Microdissection may only be possible with the use of biosurgery or maggot debridement therapy (MDT) described separately.^75^ This approach may be problematic in that every “new” injury increases the risk of complicating superinfection.^9,59,60^
(2) *Wet to dry mechanical debridement* removes nonviable tissue by allowing gauze saturated with saline and applied to a wound, to dry. The gauze then becomes adherent to the wound during the drying phase and when the gauze is removed it can nonselectively pull away both nonviable tissue along with viable granulation tissue.^3,75^
(3) *Aqueous high-pressure lavage/irrigation* involves a jet stream of saline/water that mechanically removes nonviable tissue.^75^ This is considered a nonselective form of debridement and is capable of removing granulation tissue and may pose a risk to the health care provider.^75^ The mist created by the high-pressure irrigation may expose the provider to contamination.^3,75^ Whirlpool involves a form of high-pressure hydro irrigation where the entire limb or patient is immersed in a whirlpool bath during the irrigation process.^48^
Cross-contamination is possible using this method as other wounds and body surfaces may be immersed in the same aqueous solution.^48^ This is also considered a nonselective form of debridement.^3,75^
(4) *Ultrasound debridement* utilizes sound energy to mechanically debride wounds through contact or noncontact low-frequency ultrasound energy.^75,76^The process utilizes a method of cavitation to generate sound energy from a hand-held instrument, which through mechanical means dislodges and removes devitalized tissue. Contamination to the operator/provider can also occur due to aerosolization.
(5) *Biosurgery or MDT*—This has been an area of interest for over 400 years and provides a complex system of wound care.^75^ Maggots are larva of flies, such as Lucilia Sericata that consume nonviable tissue selectively.^77^
This is typically done in the United States with another form of larva, the blow fly maggot variety (Phoenicia sericata larvae).^78^ Medicinal maggots are believed to carry out biosurgical debridement of nonviable tissue selectively compared with blunt dissection, which may reduce the risk of secondary superinfection.^78–81^
The maggots are capable of consuming bacteria and are believed to produce antimicrobial secretions.^75^ This has been demonstrated through mechanistic *in vitro* studies.^79^ MDT may have antimicrobial properties that are active against hospital acquired resistant organisms, such as MRSA.^79^ They may secrete substances that stimulate wound healing.^79^

## Standard Wound Care, and Adjunctive Prevention, and Treatment Methods

The treatment of a DFU generally involves a multidisciplinary team approach and includes comprehensive advanced wound care. This team may comprise a primary care physician, a wound care physician, a wound care nurse, a nutritionist, orthotics consultant, physical therapist, and a hyperbaracist.^3,48^ This comprehensive advanced wound care approach is endorsed through advocacy by the Alliance of Wound Care Stakeholders involving a multidisciplinary team and the following interventions.^3,48^

(1) Off-loading: Weight-bearing redistribution is the most important consideration for wound healing of the DFU. This provides support by redistribution of weight bearing away from the wound and relocates it to the adjacent surfaces of the affected foot or leg through the use of orthotics. A common error in wound care includes neglecting this critical intervention. Since sensory neuropathy perpetuates a vicious cycle of reinjury due to unrecognized trauma, offloading becomes critical in breaking this self-perpetuating cycle.^82–84^
Alternatively, complete offloading can be achieved by using wheelchairs, walkers, crutches, or other wheeled mobile devices to remove all weight-bearing entirely (nonweight bearing) from the affected wound and limb.^3^
(2) Physical therapy: The use of offloading equipment may require special instruction routinely provided by a physical therapy department in the proper use of crutches, wheelchair, or other ancillary mobile nonweight-bearing equipment.
The patient may require rehabilitation due to long periods of immobility to regain function and strength to allow for the use of offloading devices this can be done through intensive short-term inpatient rehabilitation or in an outpatient or home setting.
(3) Medical optimization of comorbidities, including diabetes: The patient requires medical optimization of current treatment for diabetes and other conditions that if left untreated or poorly controlled may impede wound healing.
(4) Nutritional consultation services and supplementation: These services have been utilized to address nutritional deficiency states that may impede wound healing. Laboratory markers, such as Total Lymphocyte Count, pre-Albumin, Albumin, and Total Protein, along with clinical parameters have been used to help direct nutritional interventions. The use of supplementation including protein supplements and micronutrients may be warranted.
(5) Infection eradication: If the wound is critically colonized or infected then this may impair wound healing and antimicrobial therapy is often prescribed. Treatment can be directed locally or systemically depending on the extent and severity of the infection.
(6) Medical and surgical vascular interventions: Hemodynamically significant macrovascular insufficiency can compound microarterial insufficiency and may require vascular surgery evaluation. Therapy may involve more extensive medical treatment, and/or the patient may require surgical revascularization, which could include angioplasty, stenting, atherectomy, or surgical bypass grafting.
(7) Hyperbaric oxygen therapy and other means of oxygen delivery: Periwound tissue hypoxia can be measured using transcutaneous oximetry. If tissue hypoxia is found to be reversible with normobaric or hyperbaric oxygen challenge, then hyperbaric oxygen therapy has been considered adjuvant therapy in healing problem refractory wounds in diabetics. This testing may reveal microvascular insufficiency. Hyperbaric oxygen therapy may increase tissue oxygen tensions up to 15 times normal. Angiogenesis and vasculogenesis may be stimulated by the using hyperbaric oxygen therapy, which may enhance the blood supply around the wound. Proinflammatory intracellular adhesion molecules are downregulated providing an anti-inflammatory effect.^85^ Edema may be decreased with the use of hyperbaric oxygen therapy through peripheral vasoconstriction without a negative effect on tissue oxygenation. Oxygen diffusion is increased up to a factor of four in the affected tissues.^86^ Antimicrobial tissue penetration and leukocyte function is believed to be enhanced by the use of hyperbaric oxygen therapy. Susceptible organisms, such as anaerobic or facultative anaerobic organisms that do not tolerate high oxygen tensions may be inhibited by using hyperbaric oxygen therapy. An increase in stem cell production, differentiation, and presence in the wound bed has been demonstrated.^87^ Hyperbaric oxygen therapy may be especially useful in those diabetics that have had wound care for greater than 4 weeks with poor or no response to advanced wound care treatment.^3,88^
Topical oxygen involves delivering oxygen over and in contact with the wound site rather than through the systemic circulation as delivered through Hyperbaric oxygen therapy.
Studies using topical oxygen delivery have been reviewed in a position statement by the Undersea and Hyperbaric Medical Society in 2005 and revised in 2018. To date, there is insufficient evidence to conclude that topical delivery of oxygen should be used in lieu of systemic hyperbaric oxygen delivery.^89^
(8) Coordination of care: This comprehensive approach includes communication between the advanced specialties for wound care (*e.g.*, surgeons, toe and flow teams, specialized DFU centers) with the respective primary care physicians and home health providers who are involved in medical optimization of the patient's health conditions, including diabetes. The accessibility in rural areas may be complicated. Telemedicine has afforded the opportunity to offset the limitation in rural regions for access to wound care professionals. Wound care professionals remotely may have visual oversight that is facilitated through the work of an onsite wound care nurse or other health care professional. Disease progression can result due to incomplete information sharing between the members of the multidisciplinary team, lost follow-ups, and patient noncompliance. The importance of intensive and close follow-up, including regular podiatric care, debridement, access to vascular in-hospital intervention to maximize the likelihood of limb salvage, is critical.^90^

## Discussion

### Mechanism of the intervention and clinical considerations in the path toward treatment

It was discussed in the section Description of the Intervention that debridement involves the removal of devitalized, contaminated, or foreign material from within or adjacent to a wound, until surrounding viable tissue is exposed and that it is widely practiced in diabetic foot care.^91^ Debridement is widely regarded by many as an effective intervention to speed up ulcer healing.

Sharp debridement of an ulcer, including the removal of callus (which may surround or “roof over” an ulceration) and devitalized tissue is viewed as an effective means to facilitate wound healing, although direct evidence of this has been lacking. Once an ulcer has developed the central aim has been to heal it in the shortest interval of time and prevent recurrence. This approach has been standard of care and has been a mainstay of treatment. For example, Edmonds^66^ suggested six aspects of “control” to be addressed when caring for people with diabetes, which are: mechanical, wound, microbiological, vascular, metabolic, and educational. Debridement is recommended by the Scottish Intercollegiate Guidelines Network (SIGN) diabetic foot guidelines alongside antibiotic therapy for infection and pressure relief as a treatment for patients who have developed ulceration or gangrene with risk of amputation.^33^ The Royal College of General Practitioners' Guidelines also recommend debridement as a treatment of the ulcerated foot alongside local wound management and appropriate dressings.^92^ Neither of the guidelines recommend a specific method of debridement; Edmonds and Foster^66^ gave the following rationale for debridement of neuropathic ulcers as it: enables the true dimensions of the ulcer to be perceived; allows for the drainage of exudate and removal of dead tissue rendering infection less likely; enables a deep swab to be taken for culture; and encourages healing. This original approach continues to be used today.

This approach has been supported. Margolis conducted a meta-analysis of the control group healing of 10 treatment trials in people with diabetic neuropathic foot ulcers and estimated that 24% heal within 12 weeks and 31% by 20 weeks with good wound care.^93^

The evidence on the competing methods of debridement has been studied in 10 systematic reviews (SRs) and meta-analyses. The conclusions do not demonstrate compelling evidence that one form of debridement is superior to other forms of debridement or to control conditions. The authors of the respective SRs report that there is weak evidence to conclude that any form of debridement is superior to any other form of debridement, including the control condition using moist gauze dressings as a control condition.

These 10 SRs included 4 to 10 studies, 6/10 SRs were restricted to randomized studies, and 4/10 SRs included both randomized and nonrandomized studies. The studies retrieved varied in quality measures. The total sample size in 10 SRs included a range of 149–575 subjects. The study follow-up period in the SR ranged from 10 days to 6 months.^59,80,94–101^

The types of wounds in the studies used in the SR were not restricted to DFUs. A total of 2/10 included venous stasis ulcers along with DFUs, and 1/10 included ischemic ulcers in addition to DFU.^95,101^

The comparisons included 1–4 methods of debridement in the studies used in the 10 SRs. These debridement methods included sharp/surgical, autolytic (hydrogel, foam, alginates, hydrocolloids, semipermeable polymeric membranes, silver-containing), larva or maggot debridement, and hydrotherapy.

The outcome measures included amputation frequency, infections rates, complete healing rates, time to complete healing, wound size reduction, health-related quality-of-life measures, wound recurrence, and adverse events.

A total of 5/10 SRs were Cochrane reviews.^59,97–100^ The SR findings on the quality of the evidence were summarized by the authors to have low evidence to no evidence that forms of nonautolytic debridement studied were beneficial. Author's conclusions in two SRs suggested moderate-to-low evidence that the reported forms of autolytic debridement were beneficial.

Meta-analyses were conducted in 6/10 studies and not conducted in 4/10 SR.^59,80,97–101^ One study included randomized and nonrandomized studies.^80^ The five Cochrane Reviews included SR that were updates of previous SRs.

The work-flow diagram below gives a general outline and broadly summarizes the approach to management of DFU. The approach ultimately relies on the discretion of wound experts and health care team to individualize the approach to the particular circumstances encountered for the individual patient.

**Work-Flow Diagram: Considerations for the Treatment and Management of the Diabetic Foot Ulcer**

**Diabetic Foot Ulcer/Wound Presentation**


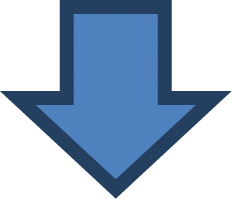


**History & Physical Examination, Comprehensive Wound Assessment including Biopsy if diagnosis unclear and wound grading or staging, Institute Immediate Steps to Offload DFU, Consider Comprehensive Team Approach, Ensure Medical Optimization of patient.**


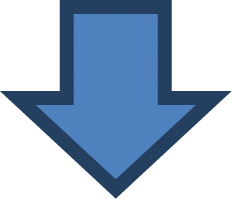


**If systemic signs/symptoms of infection/sepsis or complicated localized infection or signs/symptoms of acute/critical limb ischemia admit for inpatient emergency/acute care evaluation.**


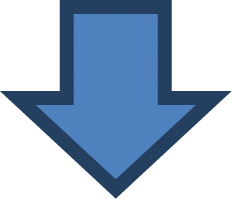


**If suspected ischemia consider: PVR/ABI studies, TcPO2, Vascular Surgery evaluation, Advanced vascular studies i.e. MRA, CT angiography, Angiogram. Consider Adjunctive Hyperbaric Oxygen Therapy.**


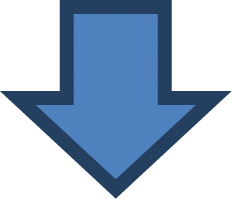


**If suspected soft tissue wound Infection/Critical Colonization consider topical/systemic antibiotics, Infectious Disease Evaluation, Surgical Evaluation if deep wound infection/abscess, bone exposed/suspected Osteomyelitis or suspected necrotizing soft tissue infection.**

**Evaluation for Osteomyelitis consider: CT, MRI, Bone Scan, or bone debridement, biopsy/pathology.**


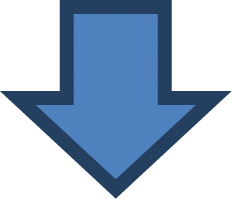


**Nonviable tissue present – Choose appropriate method of debridement & wound dressing, determine the extent of wound, comfort & stability of patient, and complexity of debridement required i.e. minor surgical debridement in ambulatory setting or intraoperative debridement.**


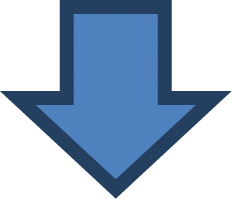


**Consider other advanced wound therapies (wound vacuum therapy, skin substitutes, collagen matrix, growth factors)**

## Conclusion

The DFU has serious consequences to the individual patient, their families, the health care system, and to society as a whole. The patients who experience amputations, and serious infections along with the associated impairment and disability results in financial hardship and lost productivity. The patient faces a reduced quality of life along with an increase in 5-year mortality. The costs of care as presented in this comprehensive review illustrates the impact this disease process has on the patient and society.

An understanding of the underlying mechanisms and pathophysiology of the disease process involving DFUs as presented in this review is critical in appreciating the value of a comprehensive scope and the rationale behind the health care team approach in preventing further complications from the DFU and their recurrence or development from the outset. This approach has important public health as well as clinical implications for successfully treating DFUs.

These tragic outcomes may be averted if efforts are made to accelerate successful wound healing. There is no standard method for debridement selection, therefore, the method used effectivity relies on the proper utilization, timing, and communication between the lead healthcare provider and those involved in DFU care to individualize the approach to treatment based on the numerous factors discussed. This includes the decision on the method of debridement used and the use of other adjunctive wound care therapies.

**Take-Home Messages**

- DFUs may lead to complications, including, but not limited to infections, amputations, disability, decreased quality of life, and death.
- DFUs are a widely prevalent problem affecting 15–34% of diabetics in their lifetime which includes a staggering number of diabetics.
- Devitalized or dead tissue promotes the production of inflammatory mediators at the biochemical, cellular, and tissue level by providing a medium for growth of infectious organisms (biofilm) impeding the migration of cells required for healing.
- Removal of devitalized (dead) tissue is known as debridement, and can be an effective modality and accomplished by a variety of methods, including mechanical and nonmechanical debridement
- Nonmechanical debridement includes autolytic (moist dressings), and enzymatic (collagenase) whereas mechanical debridement includes sharp/surgical, biosurgery (MDT), hydrotherapy/whirlpool, and ultrasound.
- Selection of these methods should be based on multiple factors that are patient-specific and provider-specific, with the goal of ultimately reducing the risks of complications such as infection, amputations, disability, decreased quality of life, death and increasing associated cost.
- Advanced wound care modalities for DFUs should result in a better quality of life for the patient and their family. The best method(s) of debridement are unclear and still under investigation.

## About the Authors

**David Dayya, DO, PhD, MPH**; Associate Medical Director, Division of Undersea and Hyperbaric Medicine—Department of Surgery, Phelps Hospital Northwell Health. Associate Professor, Division of Undersea and Hyperbaric Medicine—Department of Emergency Medicine, State University of NY—Upstate Medical University. Clinical Assistant Professor, Department of Family Medicine—University of Vermont College of Medicine. **Owen J. O'Neill, MD, MPH, FUHM**; Founding Medical Director and Chief, Division of Undersea and Hyperbaric Medicine—Department of Surgery, Phelps Hospital Northwell Health. Associate Professor, Division of Undersea and Hyperbaric Medicine—Department of Emergency Medicine, State University of NY—Upstate Medical University. Assistant Professor, Department of Internal Medicine—New York Medical College. **Tania B. Huedo-Medina, PhD, MS**; Associate Professor, Biostatistics—Department of Allied Health Sciences—University of Connecticut. **Nusrat Habib, MBBS, MS**; Research Assistant, Department of Allied Health Sciences—University of Connecticut. **Joanna Moore, MD**; Resident Physician, Department of Internal Medicine, Norwalk Hospital, Nuvance Health System. **Kartik Iyer, MD**; Resident Physician, Department of Family Medicine, Hackensack—Meridian Hospital, John F. Kennedy Health System.

## Abbreviations and Acronyms

ABI: ankle-brachial index
DFU: diabetic foot ulcer(s)
DM: diabetes mellitus
IDF: International Diabetes Federation
IWGDF: International Working Group on the Diabetic Foot
MDT: biosurgery or maggot debridement therapy
MRA: magnetic resonance angiogram
MRSA: methicillin-resistant *Staphylococcus aureus*
NHS: National Health Service
PAD: peripheral arterial disease
PVR: pulse volume recording
SPP: skin perfusion pressure
SR: systematic review
TcPO_2_: transcutaneous partial pressure of oxygen/transcutaneous oximetry
TMA: transmetatarsal amputation
TP: toe pressure
Wifi: wound, ischemia, foot infection (Society of Vascular Surgery threatened limb ischemia classification system.)
